# Aquatic quillworts, *Isoëtes echinospora* and *I. lacustris* under acidic stress—A review from a temperate refuge

**DOI:** 10.1002/ece3.9878

**Published:** 2023-03-08

**Authors:** Martina Čtvrtlíková, Jiří Kopáček, Jiří Nedoma, Petr Znachor, Petr Hekera, Jaroslav Vrba

**Affiliations:** ^1^ Biology Centre CAS, Institute of Hydrobiology České Budějovice Czech Republic; ^2^ Faculty of Science Palacký University Olomouc Olomouc Czech Republic; ^3^ Faculty of Science University of South Bohemia České Budějovice Czech Republic

**Keywords:** acidification, isoetids, reproduction, softwater lakes, toxicity

## Abstract

Quillworts (*Isoëtes*) represent highly specialized flora of softwater lakes, that is, freshwater ecosystems potentially sensitive to acidification. In this paper, we combine a review of previous studies and our new results to address unrecognized reproduction strategies of quillworts to overcome long‐term environmental stresses. These strategies play an important role in the plant's ability to overcome atmospheric acidification of freshwaters, protecting the plants until their environment can recover. Environmental drivers of recovery of *Isoëtes echinospora* and *I. lacustris* were studied in two acidified lakes in the Bohemian Forest (Central Europe). Both populations survived more than 50 years of severe acidification, although they failed to recruit new sporelings. Their survival depended entirely on the resistance of long‐living adult plants because the quillworts do not grow clonally. During the past two decades, a renewal of *I. echinospora* population inhabiting Plešné Lake has been observed, while no such renewal of *I. lacustris,* dwelling in Černé Lake, was evident, despite similar changes in water composition occurring in both lakes undergoing advanced recovery from acidification. Our in vitro experiments revealed that the threshold acidity and toxic aluminium concentrations for sporeling survival and recruitment success differed between *I. echinospora* (pH ≤ 4.0 and ≥300 μg L^−1^ Al at pH 5) and *I. lacustris* (pH ≤ 5.0 and ≥100 μg L^−1^Al at pH 5). The higher sensitivity of *I. lacustris* to both stressors likely stems from its year‐long germination period and underlines the risk of exposure to chronic or episodic acidification in recovering lakes. By contrast, the shorter germination period of *I. echinospora* (2–3 months) enables its faster and deeper rooting, protecting this quillwort from periodic acidification during the next snowmelt. Our study brings novel insights into widely discussed environmental issues related to the long‐term degradation of softwater lakes, which represent important hotspots of pan‐European biodiversity and conservation efforts.

## Graphical abstract

Isoëtes echinospora and I. lacustris, the survivors in acidified lakes, have in fact suffered and their recruitment of new plants failed over decades. Stress‐tolerant life strategies of the quillworts, however, remain successful in Anthropocene.

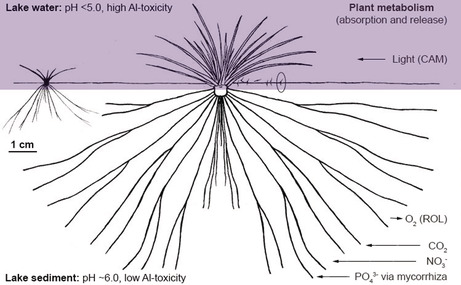

## 1 INTRODUCTION

Isoetids are acid‐tolerant plants that dominate softwater lakes, an abundant freshwater ecosystem in the Northern hemisphere (Murphy, 2002; Rørslett & Brettum, 1989). Among isoetids, the *Isoëtes* species (quillworts; Lycophytes, Isoëtaceae) are primary aquatic plants with essentially indistinguishable fossils going back to the late Jurassic (Pereira et al., 2017; Pigg, 2001; Retallack, 1997) and ancestors originated probably by the early Devonian (>400 mil years ago, Pigg, 2001). Quillworts have developed multiple adaptive mechanisms to cope with variations in water composition including natural acidification of oligotrophic softwater lakes (Madsen et al., 2002; Smolders et al., 2002). They also may have evolutionary experiences with severe acidification exacerbated by additional environmental pressures presumably not involved in the convergent evolution of other isoetids.

Quillworts were among the survivors of three major mass extinctions, associated with volcanically driven acidification (Black et al., 2014; Van de Schootbrugge et al., 2009) accompanied by metal poisoning (Chu et al., 2021; Lindström et al., 2019; Rakociński et al., 2020) and climate changes (Bond et al., 2015; Pisarzowska et al., 2020; Racki, 2020; Zhang et al., 2021). As evident from their adaptive radiations and worldwide distribution, quillworts (and other lycophytes) have up to now found opportunities in these crises (Looy et al., 2021; Pigg, 2001; Retallack, 1997; Van de Schootbrugge et al., 2009). One example, among many serious threats, is from the Permian–Triassic transition when the Northern Hemisphere received sulfur‐rich volcanogenic rainfall with annually averaged pH ≈ 2–3 during multiple eruptions of Siberian Traps lasting <10 k.y. within 1 m.y. and affecting the acidity of rain (pH ≈ 4) globally (Black et al., 2014).

During the second half of the 20th century, further acidic stress occurred in numerous European and North American freshwater ecosystems due to high depositions of sulfur and nitrogen compounds originating from industrial and agricultural sources. In severely polluted areas, precipitation pH dropped to 4.0 and the carbonate buffering system was depleted for decades, causing severe acidification of sensitive (weakly buffered) softwater lakes (Kopáček et al., 2016; Moldan et al., 2013; Skjelkvåle et al., 1998; Stoddard et al., 1999; Wright et al., 2005). While numerous original biota failed to survive, quillworts and other isoetids persisted in many of the acidified lakes (Farmer, 1990; Murphy, 2002; Rørslett & Brettum, 1989; Vrba et al., 2003). It is, however, unclear whether and how the isoetids can withstand continuing atmospheric acidification and whether some further human‐induced stress (e.g., eutrophication or climate change) may contribute to isoetid disappearance from still acidified (even though currently recovering) lakes.

The reproductive success of the extant submerged aquatic quillworts depends exclusively on crossing advantages of heterospory, as vegetative propagation is apparently rare (Eames, 1936), and scarcely reported in some amphibious species (Bray et al., 2018; Caldeira et al., 2021). Our research of *Isoëtes echinospora* Durieu and *I. lacustris* L. in severely acidified Bohemian Forest lakes (Czechia) gave the first compelling evidence that these isoetids, seemingly thriving under acidic conditions for more than half a century (Vrba et al., 2003), have in fact suffered. Recruitment of new plants failed although vital adult populations of both quillworts have persisted without any other adverse symptoms (Čtvrtlíková et al., 2009, 2016, 2020). These findings raised further questions about how long the adult plants can sustain unsuitable conditions in acidified lakes and whether eventual delays in recruitment may have adaptive value in terms of stress avoidance (in addition to acid tolerance).

Recent studies on several herbaceous plants suggest that reproductive timing (phenology) can vary in response to environmental conditions (Chevin & Lande, 2011; Reed et al., 2011; Yeh & Price, 2004). These changes (temporal plasticity) appear to directly affect plant fitness and, in turn, may be essential for population persistence in novel environments and in response to natural selection (Forsman, 2015; Sultan, 2000). However, neither general patterns of the quillworts' reproductive phenology nor possible temperature‐related plasticity in a wide distribution area is fully understood.

The fact of amazingly long‐lasting persistence of *Isoëtes echinospora* Durieu and *I. lacustris* L. observed in the acidified Bohemian Forest lakes under conditions of their unsuccessful recruitment was our motivation to investigate the adaptive traits that allow quillworts to sustain chronic acidic stress and to identify the survival bottlenecks that would increase the risk of their potential extinction. Here, we synthesize the main results of 20‐year research on the effects of low water pH, and consequently elevated aluminium (Al) toxicity on these isoetids, clarifying the causal links between their species‐specific phenology and distinct patterns enabling their recovery after the relief of acidic stress (Čtvrtlíková et al., 2009, 2012, 2014, 2016, 2020).

Unlike lakes of Atlantic lowlands (Arts, 2002; Brouwer et al., 2002; Roelofs et al., 2002) or acidified Boreal‐Atlantic lakes under liming management (Brandrud, 2002; Lucassen et al., 2016), the Bohemian Forest lakes were not anthropogenically eutrophised. Thus, we could examine the direct acidification effects (under otherwise natural conditions) on these highly specialized isoetids that have been rarely reported in the literature. This comprehensive overview of quillworts' adaptability to severe and long‐lasting acidification stress is based on our scientific knowledge, long‐term field observations, and new experimental work. Our motivation is to sum up an available spectrum of ecological and evolutionary inquiry for future research on isoetids and their fate under changing environmental conditions.

## 2 STUDY SITES AND HISTORY OF THEIR ATMOSPHERIC ACIDIFICATION

**FIGURE 1:**
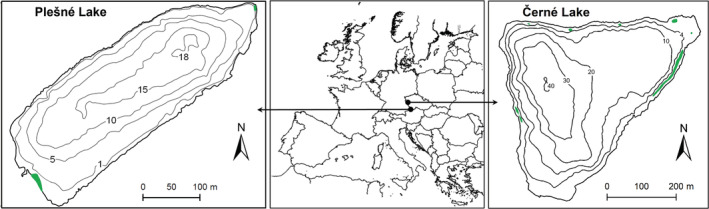
Locations of *Isoëtes echinospora* in Plešné Lake and *I. lacustris* in Černé Lake (green areas) and their position in Europe.

The impacts of acidity and Al toxicity on *I. echinospora* and *I. lacustris* were studied in severely acidified Plešné and Černé lakes, respectively (Čtvrtlíková et al., 2009, 2016, 2020). The lakes are situated in the Bohemian Forest (Šumava Mountains) at the border between Czechia, Austria, and Germany (13–14 °E, ~49 °N) at elevations of 1089 (Plešné) and 1008 (Černé) m (Figure 1) and are the only sites in Czechia inhabited by these isoetids. Both lakes are of glacial origin, dimictic, with water residence times ~350 and ~600 days, respectively (Table 1), and represent central European mountainous refuges, possessing biogeochemistry similar to the Boreal strongholds of the quillworts (Arts, 2002).

**TABLE 1:** Main geographical and morphological characteristics of Plešné and Černé catchment‐lake systems (Šobr & Jánský, 2016)

Characteristics	Unit	Plešné	Černé
Longitude (WGS)	°E	13.867	13.183
Latitude (WGS)	°N	48.783	49.183
Lake elevation	m	1087	1008
Lake area	ha	7.2	18.8
Maximum depth	m	17.7	40.1
Mean depth	m	7.7	15.6
Lake volume	10^3^ m^3^	553	2925
Catchment area^a^	ha	67	124
Maximum elevation	m	1378	1343
Mean inclination	degree	12	14
Soil^b^	kg m^−2^	92	225
Bedrock		granite	mica schist

^a^ Including lake area.

^b^ Average bulk density of fine soil (cambisols and haplic podzols; <2 mm, 105°C dried) was calculated as area‐weighted mean (Kopáček et al., submitted).

**FIGURE 2:**
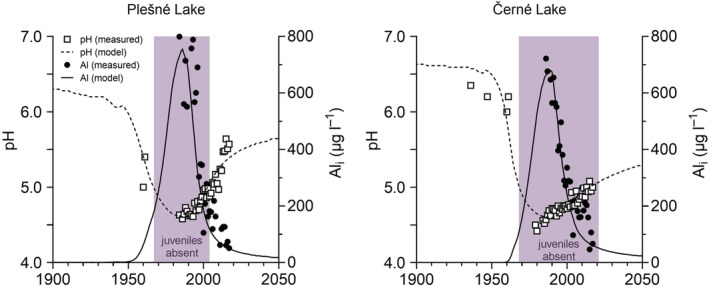
Long‐term trends in water pH and concentrations of ionic aluminium (Al_i_) in Plešné and Černé lakes. Lines are based on modeling; symbols are observed data (Majer et al., 2003). Quillwort juveniles were probably absent for decades in both lakes (violet area) as evident from the field observations and laboratory experiments on threshold acidity and Al toxicity for sporeling survival (Čtvrtlíková et al., 2009, 2020).

Plešné Lake is mesotrophic, with average total phosphorus (P) and chlorophyll *a* concentrations of 16 and 41 μg L^−1^, respectively, while Černé Lake is oligotrophic with the present average concentrations of 3.5 μg L^−1^ P and 3 μg L^−1^ chlorophyll *a* (Table 2). The primary production of lakes is P‐limited (Kopáček et al., 2011). Anoxia occurs regularly only in the bottom layer of Plešné Lake (below 14 m depth) during both the winter and summer stratification periods (Kopáček et al., 2004). The studied lakes belong to a softwater category, with low conductivity, acid neutralizing capacity (ANC), and concentrations of both calcium (Ca) and magnesium (Mg) (Table 2). The lakes were already atmospherically acidified in the early 1960s (pH < 5.4; depleted carbonate buffering system), and water acidification progressed until the middle 1980s when their pH dropped to 4.4–4.8, and Al concentrations exceeded 1 mg L^−1^ (Veselý et al., 1998). The ionic forms of Al (Al_i_, i.e., the sum of mostly Al^3+^ and Al‐OH complexes) represent the dominant form of total Al in lake water and the major toxicant for in‐lake biota (Figure 2 **)**.

**TABLE 2:** Major water characteristics (arithmetical averages of all available data) of Plešné and Černé lakes (Kopáček et al., 2017; Majer et al., 2003; Veselý et al., 1998)

Characteristic^a^	Unit	Plešné Lake				Černé Lake			
		1980s	1990s	2000s	2010s	1980s	1990s	2000s	2010s
Transparency	m	ND	2.0	1.2	0.9	9.5	6.9	7.9	6.8
pH		4.6	4.7	4.9	5.2	4.6	4.8	4.8	5.0
Conductivity	μS cm^−1^	35	27	22	20	37	29	23	21
SO_4_	mg L^−1^	7.7	5.7	3.3	2.2	6.8	4.5	3.1	2.5
NO_3_‐N	mg L^−1^	0.30	0.35	0.64	0.72	1.23	0.93	0.87	0.82
Cl	mg L^−1^	0.54	0.52	0.49	0.43	0.96	0.67	0.59	0.54
Na	mg L^−1^	0.90	0.96	0.90	0.89	0.77	0.86	0.73	0.71
K	mg L^−1^	0.41	0.39	0.48	0.78	0.53	0.47	0.46	0.52
Ca	mg L^−1^	1.44	1.00	0.85	0.94	0.98	0.89	0.72	0.69
Mg	mg L^−1^	0.32	0.22	0.19	0.23	0.56	0.47	0.40	0.42
NH_4_‐N	mg L^−1^	0.06	0.05	0.07	0.04	0.04	0.05	0.05	0.03
DOC	mg L^−1^	1.6	3.5	4.0	6.1	ND	1.4	1.7	1.9
TON	mg L^−1^	ND	0.38	0.48	0.64	0.24	0.18	0.22	0.25
TP	μg L^−1^	7.5	10.0	11.7	15.8	4.1	3.6	3.2	3.5
Al	μg L^−1^	933	608	525	501	787	461	275	235
Al_i_	μg L^−1^	ND	358	246	170	ND	314	203	150
ANC (Gran)	μmol L^−1^	−17	−17	−10	7	−22	−16	−13	−5
Chlorophyll *a*	μg L^−1^	ND	9	21	40	2	3	2	3

Abbreviation: ND, not determined.

^a^ Transparency was measured as Secchi depth; average pH = −log(average H^+^ concentration); conductivity was measured at 25°C; concentrations of sulphate (SO_4_), nitrate‐nitrogen (NO_3_‐N), chloride (Cl), sodium (Na), K, Ca, Mg, and ammonium‐nitrogen (NH_4_‐N) were measured by ionic chromatography (metals alternatively also by atomic absorption or inductively coupled plasma spectrophotometry); DOC was measured in the filtrate (glass‐fiber filters, pore size of 0.4 μm) after combustion as CO_2_ with different types of C‐analyzers; total organic nitrogen (TON) was the difference between the Kjeldahl N (determined by Kjeldahl digestion according to Procházková, 1960) and NH_4_‐N; total phosphorus (TP) was determined by perchloric acid digestion and the molybdate method (Kopáček & Hejzlar, 1993); concentrations of total Al and Al_i_ were determined according to Driscoll (1984); ANC was determined according to Gran (1950); and chlorophyll *a* was determined spectrophotometrically on Whatman GF/C filters after acetone extraction (Lorenzen, 1967) without corrections for phaeopigments. The accuracy of the analytical results was controlled by balancing ionic charges (including concentrations of organic acid anions and Al_i_; Kopáček et al., 2000). The differences between the sums of all cations and anions were <±5% of the total ionic concentration in the individual water samples used in this study since the 1990 s. For more details on methods and their detection limits, see Kopáček et al. (2017).

At present, the lakes have been recovering from acidification after an unprecedented reduction in sulfur and nitrogen emissions in central Europe since the late 1980s (Majer et al., 2003). For example, in Czechia, the present emissions of sulfur and the sum of oxidized and reduced nitrogen forms are >90% and >65% lower than their maxima in the 1980s and decreased to their levels typical for the 1870s and 1940s, respectively (Kopáček et al., 2016). The biological recovery of the lakes is delayed after their chemical reversal, being still fishless and with slowly recovering plankton and littoral communities (Čtvrtlíková et al., 2016, 2020; Vrba et al., 2016).

The lake catchments are steep corries with slowly weathering bedrock and are covered with shallow acidic soils (Table 1). Wetlands and bare rocks represent a negligible proportion of their catchments, while forest vegetation occupies ~90% of their areas and is dominated by Norway spruce (*Picea abies*). Both catchments have been affected by bark beetle (*Ips typographus*) infestation (Schmidt et al., 2022). The resulting tree dieback killed >90% of mature spruce stands in the Plešné catchment between 2004 and 2008 (Kopáček et al., 2017), while the forest infestation has been continuously graduating in the Černé catchment since the 2000s. All dead biomass was left in the catchments, which are parts of the Šumava National Park. Natural forest regeneration has been fast in the affected areas due to the high availability of sunlight on the forest floor, high soil moisture, and available nutrients in soils from decomposing dead biomass (Kaňa et al., 2013, 2019).

The rapid tree dieback and release of elements from decomposing dead biomass significantly affected water composition in Plešné Lake (Kaňa et al., 2019; Kopáček et al., 2017). Elevated leaching of nitrate was accompanied by terrestrial co‐export of protons (H^+^), Al_i_, and base cations, especially Ca, Mg, and potassium (K). The temporally decreased water pH and higher Al concentrations delayed lake water recovery from atmospheric acidification for 5–10 years. About a decade after tree dieback, however, nitrate, H^+^ and Al_i_ leaching decreased, while terrestrial exports of dissolved organic carbon (DOC) and P increased (Kopáček et al., 2017, 2018). The in‐lake nitrate assimilation and denitrification (due to increased primary production after elevated P input) and increased photochemical and microbial oxidation of organic acid anions, associated with elevated DOC inputs, resulted in a rapid increase in internal alkalinity production in Plešné Lake (Kopáček et al., 2019). The decreasing acidic deposition and changes in terrestrial element exports after the tree dieback in the unmanaged Plešné catchment resulted in accelerating lake water recovery from acidic stress, accompanied by a re‐establishment of the carbonate buffering system (after more than half a century) and pH approaching ~5.5 (Kopáček et al., 2019).

Similar changes were less important in Černé Lake due to still slow and less pronounced tree dieback in its catchment. Hence, DOC concentrations increased only slightly, and P concentrations have remained stable in this lake, compared to their increases in Plešné Lake, especially in the 2010s (Table 2). However, even in Černé Lake, pH has already increased to ~5.0 and low, positive ANC values (<7 μmol L^−1^) have occurred occasionally in its epilimnion during summer periods since 2015 (Table 2).

## 3 EFFECTS OF LAKE ACIDIFICATION ON *ISOËTES* SPECIES IN THE BOHEMIAN FOREST LAKES

**FIGURE 3:**
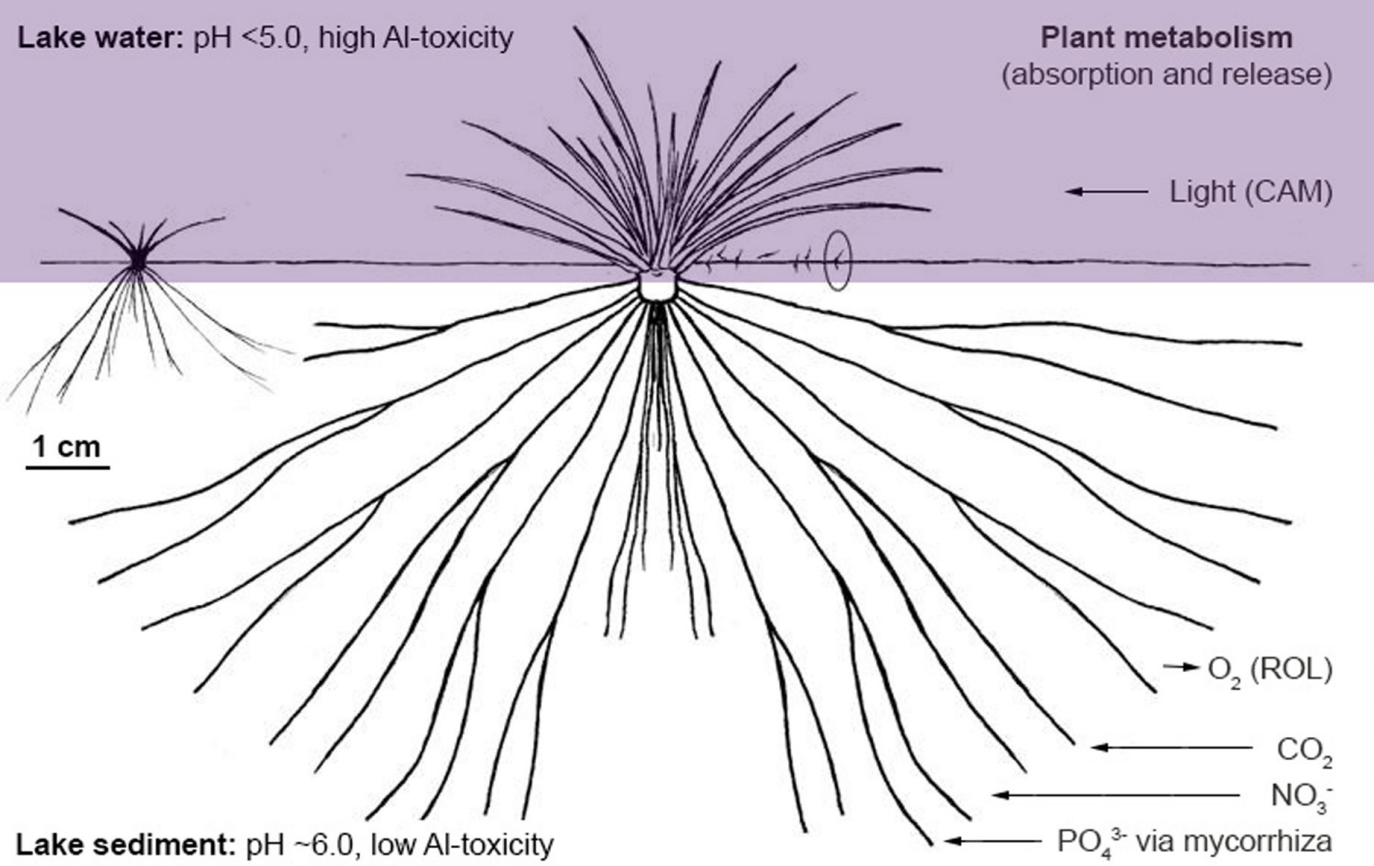
Schematic distribution of leaf rosettes (assimilative organs) and roots (absorptive organs) of adults (centre), juveniles (left), and sporelings (circle on the right) of quillworts at the water–sediment interface. The severity of potential acidification stressors (pH, Al_i_) in ambient lake water and sediment pore water is generalized on the upper and lower left. It is illustrated that the short, fine roots of early ontogenetic stages are exposed to the harsh, growth‐limiting conditions of acidic, Al‐rich lake water penetrating the sediment surface (violet area), while the long roots of adults are saved in the deeper sediment. An adult plant metabolism in terms of absorption (left arrows) and release (right arrow) sites (leaves or roots) for light (Crassulacean acid metabolism, CAM), carbon, nutrients, and oxygen (radial oxygen loss, ROL) is demonstrated on the right, following Smolders et al. (2002) and Keeley (2014).

*Isoëtes* species are the epitome of isoetid growth with a high root:shoot ratio and root‐based nutrition to exploit relatively rich sediment supplies (Rørslett & Brettum, 1989; Smolders et al., 2002; Figure 3). Small evergreen rosettes are formed by leaves covered with a thick cuticle that are only capable of light absorption and photosynthesis (Keeley, 2014) but not nutrient uptake (Madsen et al., 2002; Figure 3). All leaves are, at least potentially, sporophylls, bearing a normal or abortive sporangium ventrally close to the base. These lower parts are buried in the upper sediment and release macro‐ and microspores as individual sporangia on the outermost leaves become mature and decayed (Eames, 1936).

The reduced pH along with elevated Al_i_ concentrations during acidification of catchment‐lake systems are the most relevant changes adversely affecting aquatic macrophytes (e.g., Arts, 2002; Čtvrtlíková et al., 2009; Schindler, 1988; Smolders et al., 2002). Both stressors act as potent root toxicants with a detrimental impact on plant fitness (Ma, 2007; Rout et al., 2001). The acidic, Al‐rich lake water, however, penetrates only to the sediment surface (<1 cm layer) (Herlihy & Mills, 1986; Kelly et al., 1984; Kopáček et al., 2001), where the shallow roots of juveniles develop. It suggests that juveniles are more endangered by acidic, Al‐rich water than the more deeply rooted adult plants (Figure 3). Hence, lake water acidification affects quillwort recruitment success more than the survival of the adults. Consequently, adult populations of *Isoëtes echinospora* and *I. lacustris* remained vital over the whole acidification period, but without juveniles, while other macrophytes vanished from the Bohemian Forest lakes (Čtvrtlíková et al., 2009, 2020; Husák et al., 2000; Figure 2). The top‐heavy age structure of quillworts indicated a failure of their reproduction, exclusively relying on the fusion of male and female gametes (crossing). Thus, there was a potential risk of progressive aging or random disturbances of adults that could eventually lead to the extinction of their relict populations.

**FIGURE 4:**
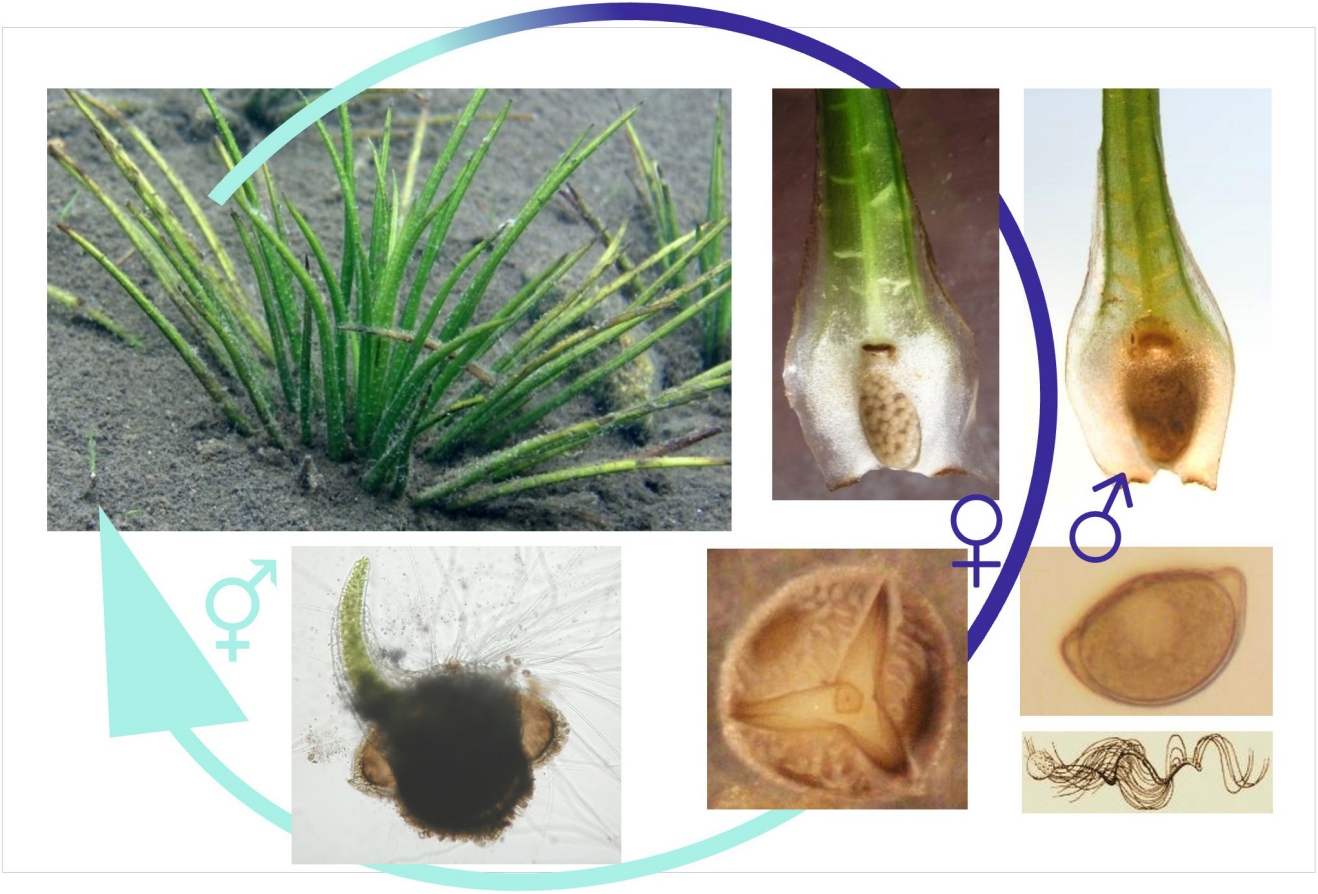
The disrupted reproduction cycle of *Isoëtes lacustris* in Černé Lake during the acidification period. Only adult plants and sporelings (at the start and the end of the arrow, respectively) occurred in the quillwort population until recently. Successful sporogenesis in sporangia on leaf bases allowed for the production of vital macrospores (♀) and microspores (♂), capable of germinating and fertilizing (spermatozoid illustration at the right lower corner taken from Eames, 1936). However, sporelings (⚥) developing in the acidic, Al‐rich water at the sediment surface did not attain the juvenile stage and perished within several months.

To identify which ontogeny stages of quillwort populations are most sensitive to acidification stress, repeated diving surveys over the whole vegetation season examined the complete reproduction cycle in situ (Figure 4). We found that adult plants of both quillwort species produced viable spores in their sporangia. These spores were able to germinate, spermatozoids were able to fertilize egg cells in gametophyte archegonia, and they even produced sporelings. We observed numerous tiny sporelings in both populations every spring. They, however, did not attain the juvenile stage and vanished by autumn for most of our study period. Based on these observations we hypothesized that adults of both species were fecund to produce viable spores **(**Figure 4), but early ontogenetic stages were vulnerable in the acidic water, causing a failure of recruitment (Čtvrtlíková et al., 2009, 2016, 2020).

**FIGURE 5:**
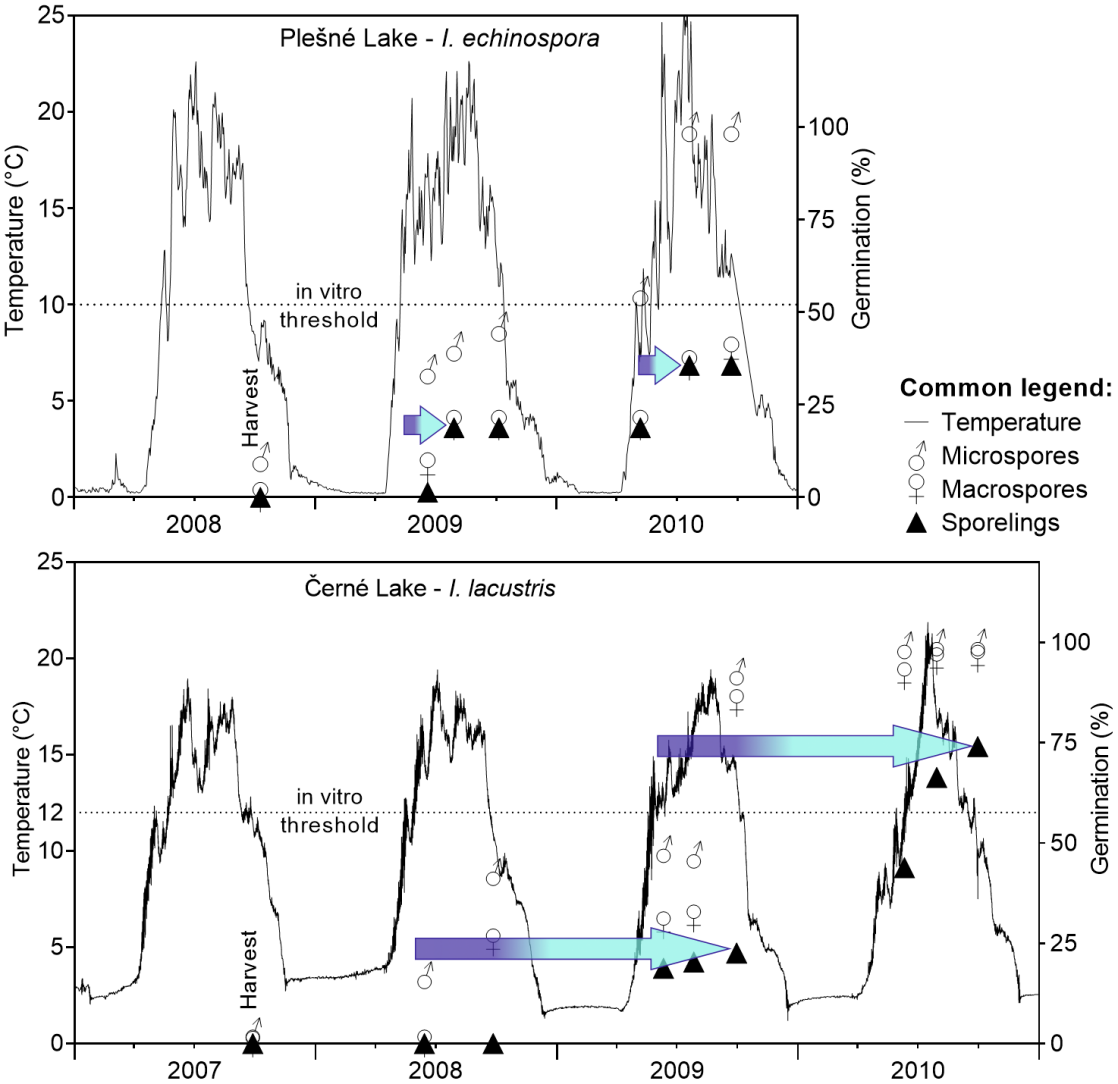
Comparison of the in situ germination rates (blue arrows) of *Isoëtes echinospora* in Plešné Lake and *I. lacustris* in Černé Lake. Macrospores and microspores harvested in autumn were grown in Eppendorf tubes at the bottom of the lakes and inspected for germination and sporeling development; symbols are means of cumulative data. Solid lines are the ambient lake water temperatures recorded continuously using a Minikin QT smart sensor (Environmental Measuring Systems Company, Brno, Czech Republic). Dotted lines indicate the temperature thresholds for spore germination derived in laboratory experiments for the same quillwort populations by Čtvrtlíková et al. (2012, 2014). Both types of spores of either quillwort species germinated gradually in cohorts over several growing seasons. The time course of individual macrospore germination and sporeling formation (schematically illustrated as in Figure 4 by the dark and light blue part of the arrows, respectively) is much shorter in *I. echinospora* (2 to 3 months) than in *I. lacustris* (minimum 1 year, always including winter). Please note that the lake experiments started in different years, but harvest seasons are aligned here for visual clarity of interspecific differences in germination rates.

To understand, what time of year is critical for the early ontogenetic stages exposed to acidic conditions, we studied the germination phenology of quillworts. These experiments investigated the effects of temperature on the spore germination of quillwort species. In the laboratory, spores were treated at temperatures between 6 and 17°C. In addition, we exposed spores in open tubes to the natural lake water temperature regimes. Both quillwort species were found to have similar temperature thresholds for germination at 10–12°C, but they differed considerably in germination length (Čtvrtlíková et al., 2012, 2014) (Figure 5). While *I. echinospora* germinated for only 2 to 3 months in early summer, *I. lacustris* needed the whole year to germinate and fully develop new sporelings. Consequently, the full germination of *I. lacustris* always included winter and snowmelt periods, when the stress conditions were most severe (i.e., lowest pH and highest Al_i_ concentrations). We also demonstrated that macrospores and microspores did not germinate all at once when released from an appropriate sporangium, but subsets of spores in a sporangium cohort germinated at intervals over several years, depending on the temperature regime (Figure 5; Čtvrtlíková et al., 2012, 2014). Such an adaptive reproduction strategy avoids the risk of germination failure in a complete spore cohort exposed to temporarily (seasonally) unsuitable conditions. However, the detrimental acidification stress was chronic over decades.

**FIGURE 6:**
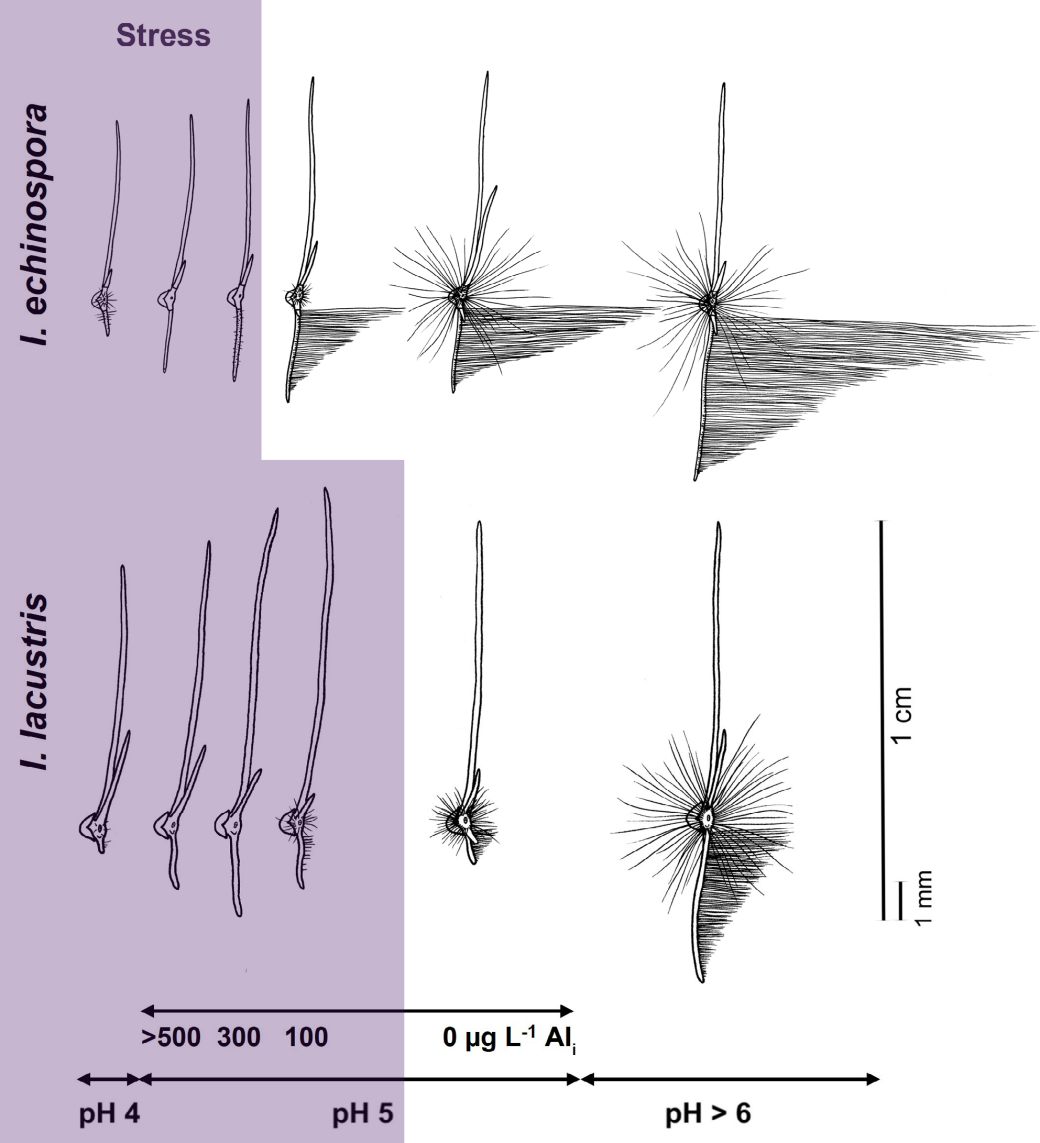
Comparative illustrations of *Isoëtes echinospora* and *I. lacustris* sporelings developed under various experimental treatments. In pH treatments (pH 4–7), the growth of absorptive (rhizoids, root, and root hairs) rather than assimilative (leaves) organs was inhibited with pH decrease. In Al‐treated variants at pH 5.0, aluminium addition (100–1000 μg L^−1^ Al_i_) reduced lengths of absorptive rather than assimilative organs. The pH = 5 variant represents the aluminium experiment's control (0 μg L^−1^ Al_i_). The threshold acidity and Al_i_ concentrations for sporeling survival differed between the species and caused different plant stress (violet area). In the diagram, sporeling dimensions are proportional among experiments. Please note that the drawings represent sporelings of the same stage (approximately two‐leaves‐two‐roots stages at the control) but different ages due to the species‐specific ontogeny (*I. echinospora* sporelings here developed from archegonia for 3 months, while those of *I. lacustris* for 18 months). For more details on methods and lake sporeling symptoms, see Čtvrtlíková et al. (2009, 2020).

To elucidate the extent to which the quillworts suffer from lake water acidification stress, we studied the direct effect of pH and Al toxicity on sporeling establishment and survival, as the fundamental prerequisites for population renewal (Čtvrtlíková et al., 2009, 2016, 2020). Laboratory experiments were performed with sporelings grown from spores of both quillworts in the gradients of pH (4–8) and Al concentrations (0–1000 μg L^−1^). Al toxicity was studied as a synergistic effect of acidic water at a constant pH of 5 and various Al concentrations. As expected, both Al toxicity and low pH reduced the size of absorptive organs of sporelings and decreased their root:shoot ratio to <1 (Čtvrtlíková et al., 2009, 2020). The dose–response relationships were found for macrogametophyte rhizoids and sporeling roots, and also root hairs were strongly inhibited (Figure 6). Moreover, the Al‐treated sporelings accumulated Al in their macrogametophytes and roots but not in leaves (Čtvrtlíková et al., 2020). Nevertheless, both the individual and/or synergistic effects of pH and Al on sporeling survival and recruitment success differed between *I. echinospora* (pH ≤ 4.0 and ≥300 μg L^−1^ Al at pH 5) and *I. lacustris* (pH ≤ 5.0 and ≥100 μg L^−1^Al at pH 5) **(**Figure 6 **)**. The higher sensitivity of *I. lacustris* to the stressors likely stems from its long germination phenology (Čtvrtlíková et al., 2014) and underlines the risk of exposure to chronic or episodic acidification in recovering lakes. Therefore, future predictions of quillwort reproduction recovery should always consider the species‐specific responses to environmental stressors and the likelihood of plants to be exposed to harsh, growth‐limiting conditions.

**FIGURE 7:**
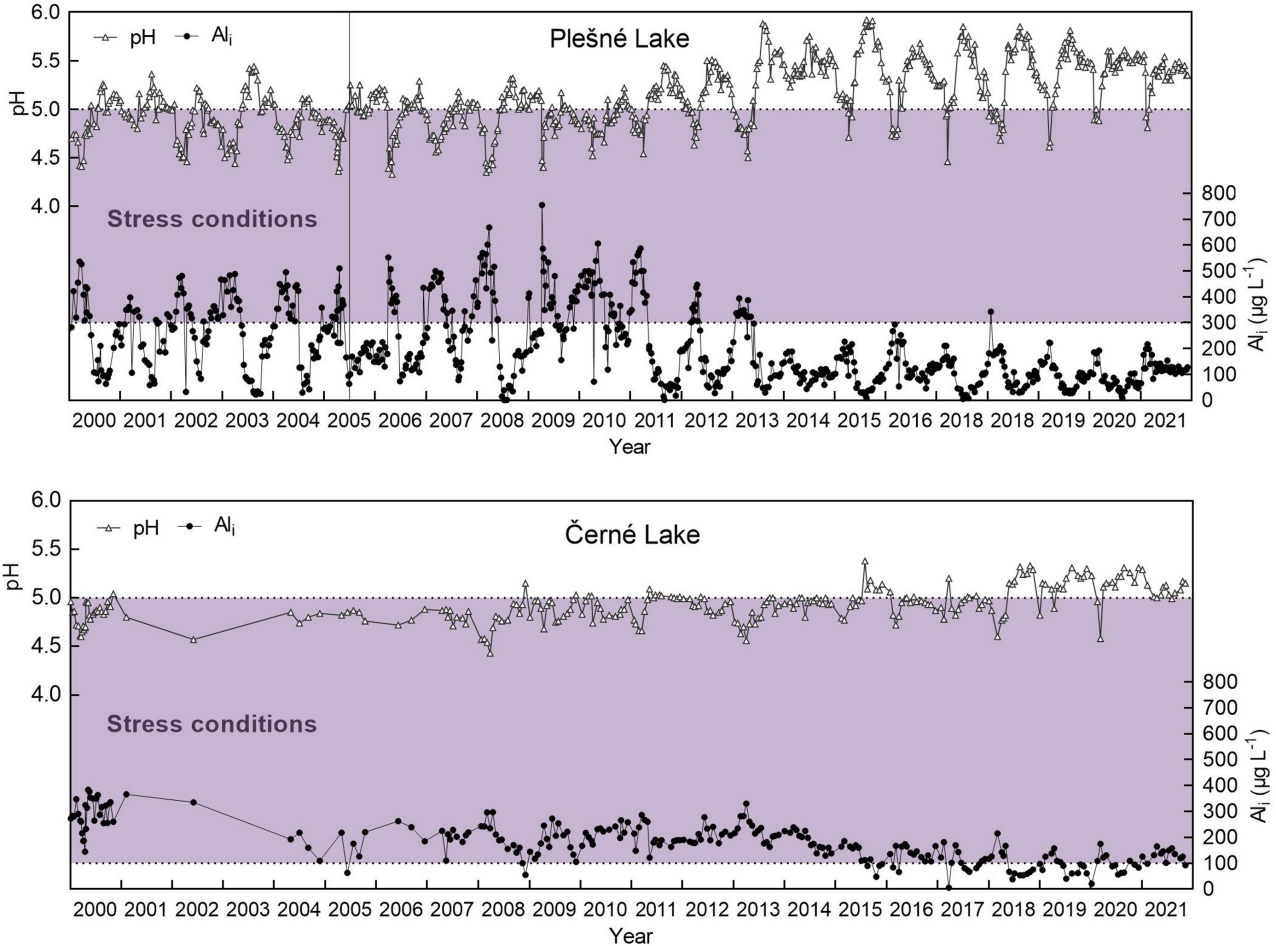
Annual variations in pH and ionic aluminium (Al_i_) concentration in the surface water of Plešné Lake and Černé Lake between 2000 and 2021. Data from Kopáček et al. (2017). Dotted lines represent threshold conditions determined in the laboratory for *Isoëtes echinospora* and *I. lacustris* early ontogeny by Čtvrtlíková et al. (2009, 2020) and delimit periods when recruitment success of the lake populations has been suppressed by the acidification stressors (violet belts). The vertical grid line shows the year when the first significant increase in adult abundance of *I. echinospora* in Plešné Lake was observed, indicating the beginning of population recovery. However, *I. lacustris* in Černé Lake has not been recovering yet.

Because lake water acidity and Al toxicity act synergistically to limit sporeling growth, we only evaluated their coupled effect on in situ populations. Thus, the individual effect of the threshold pH 4 (without Al) for *I. echinospora* recruitment was not used to assess acidification stress in Plešné Lake. Projecting the laboratory‐derived acidification stressors (threshold Al concentrations at pH 5) into the time series of the lake water chemistry (Čtvrtlíková et al., 2009, 2020), we identified the periods of extreme conditions, inhibiting recruitment of the quillworts and their effective recovery (Figure 7). Indeed, the recovery of *I. echinospora* in Plešné Lake was first observed in 2005 or could start 4 years earlier (in 2001) as reconstructed by a mathematical model (Čtvrtlíková et al., 2016). During our study, water pH increased to ≥5 and the Al concentration decreased to <300 μg L^−1^ in summer (Figure 7).

**FIGURE 8:**
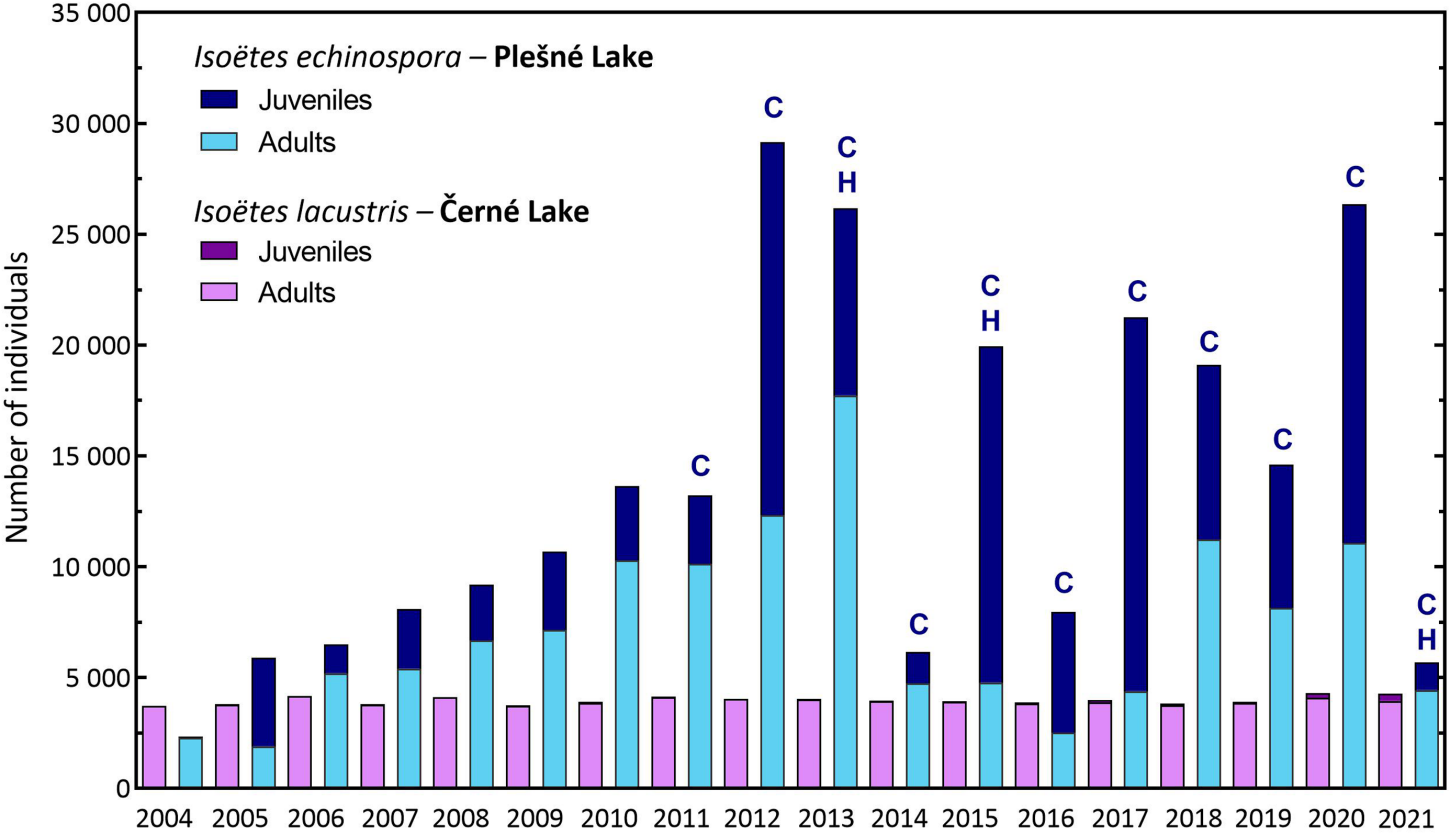
Comparison of abundances and age structures of the population of *Isoëtes echinospora* in Plešné Lake and *I. lacustris* in Černé Lake in 2004–2021. Summer (June) counts of adult and juvenile plants in both populations are shown. Recent competitive (letter “C”above bars) pressure of *Carex rostrata* and autumnal herbivory (letter “H” above bars) incidents by mallards (*Anas platyrhynchos*) and beavers (*Castor fiber*) in already recovering *I. echinospora* population result from the overall recovery of Plešné Lake. Population recovery (increase in adult plants) of *I. lacustris* has not yet started.

Obviously, the relatively fast‐germinating *I. echinospora* benefited from even the temporary summer decreases in acidity and Al toxicity, allowing for the development of great numbers of juveniles and adults in Plešné Lake (Figure 8). The stress conditions for *I. echinospora* in Plešné Lake progressively changed from all‐year‐round to episodic during the last two decades, and even have recently ceased. By contrast, a similar recovery of *I. lacustris* has not yet started in Černé Lake, even though juveniles have been observed in increasing numbers (low hundreds) discontinuously since 2017 (Figure 8). Almost all of them, however, did not survive presumably due to their shallow roots exposed to still acidic conditions in contrast to the deep root systems of adult plants. Chemical composition of deep sediment layers differs markedly from those at the water–sediment interface (Herlihy & Mills, 1986; Kelly et al., 1984). For instance, pH in the deeper sediment horizons (below 2–5 cm) remained relatively high (~5.7) despite the strong acidification of the lake water (Kopáček et al., 2001). Favorable conditions in the deep sediment thus enabled the growth of adult quillwort plants, as well as their production of viable and Al‐free spores during the long‐term acidification. Since the juveniles of *I. lacustris* develop for three or more years, sudden acidification episodes still pose a serious risk to their survival and population recovery (Čtvrtlíková et al., 2016). Because the quillworts' propagation by spores is disrupted in the phases of spore germination and sporeling growth at the water–sediment interface, only an increase in adult plants’ abundance is a reliable indicator of the *I. lacustris* population recovery (Čtvrtlíková et al., 2016).

Assuming negligible clonal growth, the survival of both quillwort populations in the Bohemian Forest relied entirely on the longevity of adult plants. The lifespan of both species has been estimated to be at least 50 years (Čtvrtlíková et al., 2009, 2016, 2020). The long lifespan of the quillworts allows population persistence even without completing the whole life cycle due to the long‐term environmental suppression of their recruitment. The archaic growth strategies of the quillworts (Rørslett & Brettum, 1989; Smolders et al., 2002) thus proved to be effective in population survival even under the conditions of atmospheric acidification—one of the most severe human impacts on water ecosystems in the modern world.

## 4 DISCUSSION: IMPLICATIONS FOR *ISOËTES* FATE DURING SOFTWATER CRISES

The acidic atmospheric pollution resulted in pH decrease and associated Al toxicity of the Bohemian Forest lakes for more than half a century. However, the other environmental conditions peculiar to softwaters (e.g., long‐lasting nutrient deficiency including inorganic carbon and increased water transparency) were not impaired to an extent that would disable quillworts' persistence. Similarly, the quillworts and other isoetids persisted, remaining competitively superior in many acidified Boreal‐Atlantic lakes while other communities massively declined (Murphy, 2002; Rørslett & Brettum, 1989). Nevertheless, Čtvrtlíková et al. (2009, 2016, 2020) documented that isoetids suffer fundamentally from the extreme lake water acidification when some of their sensitive life stages or absorptive organs are temporarily exposed to harsh conditions before they become deeply rooted in the sediment. The low pH and high Al toxicity killed many offspring generations of the Bohemian Forest quillworts, and only adults survived over decades due to rooting in deep sediments and long lifespan (Čtvrtlíková et al., 2016, 2020). However, populations that lost the ability to recruit and (re)colonize new habitats are susceptible to extinction due to additional ecosystem disturbances or further aging.

For instance, after an unintentional destruction of hundreds of *I. echinospora* plants in Plešné Lake by careless filmmakers in 1998 (Husák et al., 2000), empty places remained uncolonized until the population started to recover from acidification in the 2000s (Figure 8, Čtvrtlíková et al., 2009). Since the stress caused by water acidity has alleviated, the population of *I. echinospora* has recovered and recolonised those empty places in Plešné Lake (Čtvrtlíková et al., 2016). Interestingly, further impacts substantially reducing *I. echinospora* have been associated with later recovery or return of also another lake biota causing competition (sedge, *Carex rostrata*) and herbivory (mallard, *Anas platyrhynchos*, beaver, *Castor fiber*) stress since 2011 (Figure 8). Assuming an inverse recovery sequence, for example, that similar extensive grazing would occur currently in Černé Lake, the population of *I. lacustris* would be at the edge of extinction, because environmental constraints have not yet allowed sufficient plant reproduction. Another intriguing implication of the recruitment failure of *I. lacustris* is the location of plant stands along the vertical gradient of the lake bottom. Although Černé Lake water transparency almost doubled during the acidification period (Procházková & Blažka, 1999; Wagner, 1897; Table 2), allowing for deeper light penetration, *I. lacustris* failed to occupy the niche and remained within the preacidification depth range (Čtvrtlíková et al., 2020; Husák et al., 2000).

The stress‐induced recruitment failure could be the previously overlooked symptom that prevents quillworts and other isoetids from escaping unsuitable habitats in acidified lakes elsewhere. The mechanisms underlying man‐induced habitat disturbances, pushing the community out of the original equilibrium, can be more complex than we previously assumed. They may include additional stressors such as water level fluctuation, eutrophication, or catchment deforestation (Arts, 2002; Brouwer et al., 2002; Mjelde et al., 2013; Moldan et al., 2013) that eventually lead to changes in light availability at isoetid stands deadlocked at a fixed depth. Even an episodic decrease in light availability (e.g., due to increased water turbidity following an export of particulate matter from disturbed catchments) can limit adult plant survival (Farmer, 1990; Moeller, 1978; Rørslett, 1985, 1989). Plant uprooting, sometimes assumed as an ultimate escaping strategy, might in strongly acidified lakes result in damage to the temporarily water‐washed roots of adults, juveniles, or germinating spores, and prevent them from re‐rooting in shallow, light‐saturated habitats. The cumulative effects of disturbances may be occasionally misunderstood as the causal drivers of loss or reduced isoetid stands, while their recruitment fails primarily due to acidification. Today, we can only speculate about the causal drivers of the quillworts' loss in regions heavily polluted by acid rain in the last decades. It is, however, evident that the pH and Al toxicity thresholds for the quillwort recruitment were exceeded in many strongly acidified Boreal lakes in Fennoscandia and North America (e.g., Caines et al., 1985; Hendry & Brezonik, 1984; Raddum & Fjellheim, 1984; Sherman & Fairchild, 1994; Wright et al., 1976) and therefore could be the causal drivers of the reported local declines of quillwort populations. Moreover, the underlying extinction mechanisms could be similar also for the other isoetids due to their similar life strategies.

We demonstrate that life strategy potentially ensures quillworts' persistence when circumstances reduce their opportunities for exclusively sexual reproduction. For instance, the successive germination of spore cohorts may be a protective mechanism that allows both quillwort species to avoid short‐term vagaries of the environment by crossing and recruitment delays until favorable conditions recur. This could be particularly advantageous along the large‐scale climatic gradients of the quillworts' sympatric distribution, comprising countless softwater lakes in the Arctic, Boreal, Atlantic, and Central Europe (Murphy, 2002) where suitable thermal conditions vary largely in time (Čtvrtlíková et al., 2014). It is well known that climate primarily affects the temperature regime in lake ecosystems (George, 2010) and the temperature primarily determines the phenology of aquatic plants (Angilletta, 2009; Barko & Smart, 1981; Hutchinson, 1975). The thermal limits for the species‐specific reproduction phenology of the quillworts thus remain a challenging issue in interpreting the equilibrium state of their habitats in pristine softwater lakes and their resilience during the recently accelerating climate change. Unlike relatively uncomplicated examination of the limits for sporogenesis or germination phenology, for adult life history, it is extremely difficult due to missing evidence of plant age.

The quillwort life span remains an unresolved mystery of the stress‐tolerant life strategy and key physiological trait for predicting possible shifts from an equilibrium status or for their survival under accumulating human‐induced stress. The strict nutrient economy of quillworts results from natural selection in stressful, nutrient‐poor environments (Madsen et al., 2002; Schuurkes et al., 1986; Smolders et al., 2002) and corresponds with stress‐tolerant strategy distinguished by Grime (1977). Such a life strategy minimizes nutrient losses but results in low potential growth rates (Grime, 2002). Accordingly, quillworts are perennials with years‐long life spans, estimated up to 8–10 years by Szmeja (1994). We documented surprisingly long generation times of the two quillwort species in the strongly acidified Bohemian Forest lakes and estimated their in situ life spans of more than half a century (based on plant vitality, low mortality, and stress duration; Čtvrtlíková et al., 2016, 2020). However, the actual age of the quillworts remains a challenging gap in our understanding of the time scales of environmental variations that the taxa are exposed to and that they might tolerate to survive. The secondary growth of quillworts raises attractive suspicion of a tree‐like life span, despite the fact that they do not form distinctive rings of xylem (analogous to tree rings) (Eames, 1936). Instead, the secondary growth of quillworts is unusual, making the conclusive interpretation difficult (Campbell, 1891; Eames, 1936; Foster & Gifford, 1959). A more recent revision in the context of plant ontogeny and aging is thus highly urgent but virtually missing. Such knowledge is a crucial prerequisite for the application of modern methods of plant age measurements.

It is now more and more evident that phenological patterns may have consequences for species‐specific responses of quillworts to various habitat deteriorations, including atmospheric acidification. The distinct reproduction phenology of sympatric *I. echinospora* and *I. lacustris* could be naturally selected by environmental conditions of different niches along the depth gradient. Both quillworts can co‐occur in a lake, but *I. echinospora* prefers inhabiting the shallow littoral and *I. lacustris* the deep littoral (Bennert et al., 1999; Rørslett & Brettum, 1989). Accordingly, *I. echinospora* inhabits the shallow optimum depth of around 0.4 m in Plešné Lake and *I. lacustris* occurs deeper at around 2.5 m in Černé Lake (Čtvrtlíková et al., 2016, 2020; Husák et al., 2000). There is evidence in the literature that the distinct depth distribution pattern of the quillworts reflects gradients of plant exposure to wave actions and ice scour (Ballesteros et al., 1989; Gacia & Ballesteros, 1994; Rørslett & Brettum, 1989). The Bohemian Forest quillworts, thus, may be differently adapted to avoid the risk of leaf loss and reproduction failure due to the mechanical disturbances nearshore. Particularly, the leaf turnover of less than a year in *I. echinospora* (Rørslett & Brettum, 1989) and 2–3 years in *I. lacustris* (Gacia & Ballesteros, 1994; Kott & Britton, 1983; Rørslett & Brettum, 1989) suggests adaptation of the vertically separated species to avoid the winter freeze in time and space. The observed distinct germination phenologies of *I. echinospora* and *I.lacustris* are in accord with the differences in the leaf life span and avoidance of winter stress nearshore, having consequences for the sporogenesis timing. We therefore hypothesize that *I. echinospora* prospers in shallow habitats by evolving pliant and fast‐growing leaves, early‐germinating spores, and fast‐establishing sporelings striving to root before winter. On the contrary, *I. lacustris* inhabits the deeper littoral, and we hypothesize its stiff, slowly growing leaves and slowly establishing offspring to be the evolutionary trade‐offs between avoiding the shallow habitats and lower light availability at the depths. Our results suggest that the inherited differences in germination phenology of *I. echinospora* and *I. lacustris* also predetermined their ability to recover during the alleviating stress of atmospheric acidification. Another intriguing question to answer is whether the ploidy levels of the two species (*I. echinospora* as a diploid and *I. lacustris* as a decaploid) may have an impact on their length of time to create sporelings.

The tolerance continuum among sympatric quillworts along the depth gradient is apparent in North American lakes, where up to five quillwort species coexist and readily hybridize (Taylor & Hickey, 1992) whilst encompassed by only *I. echinospora* and *I. lacustris* in Northern Europe (Rørslett & Brettum, 1989). The distinct ecological strategies (Grime, 1977) reflecting the evolution and adaptation of quillworts across the diverse environments in the littoral (Rørslett & Brettum, 1989) may be of particular importance under recently increasing anthropogenic pressures in softwater lakes, affecting the persistence of particular species or the genera as a whole. Forming congeneric meadows across the whole littoral zone might be advantageous since the deep past of quillworts evolution.

Even though the pristine softwater lakes represent climatically controlled, fairly homogeneous, self‐contained, and supposedly resilient and stable systems nowadays (Dakos et al., 2015; Holling, 1973), various scenarios under acidification stress and additional anthropogenic pressures have been observed in the stronghold (boreal region), marginal (Atlantic lowland) or isolated relict (temperate mountainous) lakes (Arts, 2002; Baastrup‐Spohr et al., 2017; Murphy, 2002; Rørslett & Brettum, 1989). Unlike the Atlantic lowland lakes, requiring human‐assisted restorations to return isoetids (Brouwer et al., 2002), the disturbed Boreal lakes tend to recover their original state once relieved from the disturbance (Moldan et al., 2013; Skjelkvåle et al., 1998; Stoddard et al., 1999; Wright et al., 2005). However, their relatively higher resilience can be eroded to the degree that an additional, relatively subtle change may push the system beyond the tipping point (Holling, 1973) and drive isoetids to extinction. Because virtually no other macrophytes survived periods of severe acidification (Arts, 2002; Brouwer et al., 2002; Maessen et al., 1992; Rørslett & Brettum, 1989), we argue that the affected boreal softwater lakes may shift to an alternative state characterized by simplified community structure without any macrophytes if exposed to additional stress endangering quillworts or isoetids at all.

## 5 CONCLUSIONS

In this review, we present evidence from strongly atmospherically acidified but otherwise pristine temperate refuges of quillworts, whose relatively high resilience and capacity to survive have been affected by low pH and high Al toxicity. Populations of *I. echinospora* and *I. lacustris* neither increased nor completely collapsed at the edge of extinction. Instead, they have persisted through the severe stress and shifted their age structure towards the oldest cohort of adult plants. The long generation time of the adult plants suggests that the boreal lakes or temperate refuges have a high capacity to absorb adverse changes and prevent the extinction of these quillworts. However, once the ecosystem resilience has been eroded by another stress, the probability of their extinction increases. Therefore, pristine softwater lakes in remote stronghold areas, headwaters, or even isolated temperate refuges should be protected as natural reserves. Such an administrative step seems to be important for maintaining isoetid diversity hotspots and for minimizing the negative impacts of anthropogenic activities on their ecosystems, even when indications of isoetid stress has not yet been evident at the first glance.

## AUTHOR CONTRIBUTIONS

**Martina Čtvrtlíková:** Conceptualization (equal); funding acquisition (equal); investigation (equal); writing – original draft (lead). **Jiří Kopáček:** Conceptualization (equal); funding acquisition (equal); investigation (equal); writing – original draft (supporting). **Jiří Nedoma:** Formal analysis (equal); methodology (equal); writing – review and editing (equal). **Petr Znachor:** Methodology (equal); writing – review and editing (equal). **Petr Hekera:** Methodology (equal); writing – review and editing (equal). **Jaroslav Vrba:** Funding acquisition (equal); supervision (equal); writing – review and editing (equal).

## CONFLICT OF INTEREST STATEMENT

The authors declare that they have no known competing financial interests or personal relationships that could have appeared to influence the work reported in this paper.

## Data Availability

Data are available at the DRYAD database with doi.org/10.5061/dryad.nvx0k6dx2.
